# Seasonal Effects and Heritability of Litter Size at Birth and Weaning in Commercial Rabbits in Central Mexico (2015–2021)

**DOI:** 10.3390/vetsci12111040

**Published:** 2025-10-29

**Authors:** G. Manuel Parra-Bracamonte, Luis Becerril-Martínez, Fernando Sánchez-Dávila, Sherezada Esparza-Jiménez, Benito Albarrán-Portillo, Anastacio García-Martínez, Nicolás López-Villalobos, José F. Vázquez-Armijo

**Affiliations:** 1 Centro de Biotecnología Genómica, Instituto Politécnico Nacional, Reynosa 88710, Tamaulipas, Mexico; gparra@ipn.mx; 2 Centro Genético Porcino “La Concepción”, Jiquipilco 50830, México, Mexico; luis.b.m.coyo@gmail.com; 3 Facultad de Agronomía, Universidad Autónoma de Nuevo Léon, Gral. Escobedo 66050, Nuevo León, Mexico; fernando_sd3@hotmail.com; 4 Centro Universitario UAEM Temascaltepec, Universidad Autónoma del Estado de México, Temascaltepec 51300, México, Mexico; sesparzaj@uaemex.mx (S.E.-J.); balbarranp@gmail.com (B.A.-P.); agarciama@uaemex.mx (A.G.-M.); n.lopez-villalobos@massey.ac.nz (N.L.-V.); 5 School of Agriculture and Environment, Massey University, Palmerston North 4442, New Zealand

**Keywords:** rabbit, litter size, heritability, seasonality, weaning rate

## Abstract

**Simple Summary:**

Rabbit farming is an important source of meat, but a doe’s ability to produce and raise healthy young can be strongly influenced by weather conditions and farm management. In this study, we analyzed 770 litters from a commercial farm in central Mexico between 2015 and 2021 to assess how the year and season of birth affected the number of kits born alive, the number weaned, and the role of genetics. We found that the number of live-born kits varied significantly by year and season—being higher during wetter and semi-dry periods compared to the dry season, and notably greater in 2020–2021 than in earlier years. The number of kits weaned also varied between years but was less clearly linked to season, and survival rates to weaning showed only small seasonal differences. Genetic influence on these traits was very low, suggesting that improvements in farm management and mitigation of heat and drought stress are likely to yield faster and more substantial gains than selective breeding alone. These findings can help farmers and advisors prioritize practical strategies to increase productivity and reduce losses.

**Abstract:**

Reproductive performance in rabbits is highly sensitive to seasonal environmental variation and management practices, while the proportion of variance attributable to additive genetics for litter-level traits is typically low. The objective of this study was to evaluate the effects of year and season on litter size at birth (BR), litter size at weaning (WR), and weaning rate (WT), and to estimate the heritability of these traits in a commercial rabbit farm. A total of 770 kindling events recorded between 2015 and 2021 were analyzed. The mixed model for BR included the fixed effects of year and season, and the random effects of sire and residual error. The model for WR included the same structure, with BR added as a covariate. Least-squares means for fixed effects were used for pairwise comparisons using Tukey’s test. Year and season effects were significant for BR (*p* < 0.005), and the year effect was also significant for WR (*p* < 0.021). Litter size at birth ranged from 7.80 (dry season) to 9.21 (year 2020), with higher means observed during the semi-dry (8.52) and humid (8.56) seasons compared to the dry season (7.80). Litter size at weaning varied between 4.65 and 5.81 kits depending on the year. Weaning rate showed interannual variation (56.1–68.2%), but seasonal differences did not reach statistical significance (*p* < 0.075). Heritability estimates from the sire variance component were low: 0.01 for BR, 0.04 for WR, and 0.05 for WT. These results indicate that phenotypic variation in prolificacy in this population was predominantly driven by interannual and seasonal environmental factors, as well as perinatal management practices, while the additive genetic contribution was marginal.

## 1. Introduction

Rabbit meat production provides an efficient, small-scale source of high-quality animal protein for many temperate and subtropical regions and remains an important component of mixed and family farming systems where resources and land are limited [1]. Female reproductive efficiency—particularly kindling rate, litter size and pre-weaning survival—is a key determinant of herd productivity and economic return in meat rabbit systems, because prolificacy determines the number of marketable animals produced per doe per year [1]. Rabbit reproduction is highly sensitive to environmental conditions (ambient temperature, humidity, and photoperiod) because rabbits have limited evaporative cooling capacity and rely heavily on vascular ear heat exchange for thermoregulation [2]; therefore, thermal stress can reduce conception, increase embryonic mortality, and negatively affect kit survival and growth [3,4].

Worldwide, rabbit meat production has shown fluctuating trends over the last decade and is strongly concentrated in a few countries; large producers (notably China) account for a major share of global output, while production in several traditional European producers has fallen in recent years [5,6]. Estimates from international databases and recent reviews show that global rabbit meat production is substantially lower than the historical peak of the 2010–2014 period and that production is geographically concentrated, which has implications for global supply and for the comparability of productivity metrics across regions [5,7]. These facts emphasize the need to situate local productivity studies within a global context when interpreting temporal and seasonal effects.

Rabbit meat is recognized for a favorable nutritional profile—high protein content, low total fat and cholesterol, and a high proportion of unsaturated fatty acids—features that have supported its positioning as a healthful red-meat alternative in nutrition and consumer studies [5,8]. Despite these positive attributes, consumer acceptance and market integration vary markedly among countries; this heterogeneity influences the commercial scale of production and the resources available for genetic improvement programs [9,10].

From a production-biology perspective, seasonal variation in reproductive performance results from both direct physiological effects of environmental stressors (heat, humidity, and photoperiod) and indirect effects mediated by changes in feed intake, male fertility—particularly semen quality—milk yield, and management routines [2]. These effects combine to produce predictable seasonal patterns in reproductive output, including the numbers of kits born and successfully weaned in many climates [11,12]. Characterizing local seasonal patterns—and their interaction with management and housing—is therefore central to designing targeted management and mitigation strategies (e.g., cooling/ventilation, nutritional adjustments, timing of breeding) that minimize reproductive losses and stabilize supply [2,4,12].

In rabbits, litter-level reproductive traits (e.g., total born, born alive, or weaned per litter) generally exhibit low additive heritability (h^2^), indicating that most of the observed variation is attributable to environmental factors and non-additive genetic effects, rather than to additive genetic variance that can be exploited through classical selection [13,14,15,16]. However, heritability estimates reported in the literature are sensitive to factors such as population structure, statistical model (e.g., animal vs. dam models, inclusion of permanent environmental effects), and the stage of measurement—heritability of the decreases from birth to weaning [17,18]. Recent studies on genetic parameters and selection experiments confirm the predominance of environmental variance in survival and litter size traits, but also demonstrate how carefully specified models can enhance estimate accuracy and inform decision-making regarding genetic selection versus management interventions [17,19].

In Mexico, rabbit meat production reached approximately 4520 t in 2023 [20], with over 50% concentrated in the central region, where farming systems are predominantly small- to medium-scale [21,22]. This region is subject to pronounced seasonal and inter-annual climatic variability, transitioning between dry, semi-dry, and humid to temperate periods. Such fluctuations can significantly affect female reproductive performance and preweaning survival [21,23]. Incorporating localized climatic classifications or seasonal indicators into mixed-model analyses improves the identification of high-risk periods for productivity and enables more targeted implementation of mitigation measures (e.g., environmental control, nutritional strategies, and timing of breeding) [24]. Comparable concerns about seasonal stress and fertility have been reported in Mediterranean and North African rabbit production systems, yet analyses tailored to Latin American climates remain limited [25].

In commercial rabbit populations, breed composition and crossbreeding strategies influence both average reproductive performance and sensitivity to environmental factors [26,27]. In Mexico, New Zealand White (NZW), Californian (CA), and their F1 or F2 crosses are widely used in commercial meat production due to their prolificacy, growth rate, and carcass quality [28]. However, local adaptation, genetic background, and crossbreeding history vary across farms and regions, and breed-specific responses to seasonal variation can affect both average litter size and the variance components underlying heritability estimates.

The objective of the present study was to quantify year and season effects on litter size at birth (BR) and at weaning (WR), and on weaning rate (WT), and to estimate the heritabilities of these traits in a commercial rabbit farm in central Mexico during 2015–2021.

## 2. Materials and Methods

### 2.1. Farm, Records, and Experimental Unit

Records from 770 kindling events (litters) occurring between 2015 and 2021 were collected on a commercial rabbit farm in Jiquipilco, México, Mexico (19°34′18.777” N 99°43′3.727” W). The experimental unit was the litter. For each kindling event, the following variables were recorded: doe identification, sire identification (when available), kindling date, number of kits born alive per litter (BR), number of kits weaned per litter (WR), and the weaning rate (WT), calculated as WT = (WR/BR) × 100. Stillborn and mummified kits were excluded from BR.

### 2.2. Genetic Type

The animals were commercial crossbreds. The breeding nucleus primarily used crossbred does mated with New Zealand White (NZW) bucks. Therefore, the population is best characterized as commercial crossbreds with a major contribution from NZW genetics.

### 2.3. Seasonal Classification and Year Effect

Season of kindling was assigned using Pro.Clima v1.0 [29] (based on farm coordinates and kindling date), which classifies each litter into localized climatic categories—Dry, Semi-dry, or Humid—according to program outputs [29]. Each category classifies the local monthly season based on an aridity index that incorporates rainfall and evapotranspiration, the latter estimated using temperature and solar radiation data [29]. The year effect corresponded to the calendar year of kindling (2015–2021).

### 2.4. Productive and Reproductive Management

The farm operated a five-week batch production system, consisting of four weeks for gestation and one week for kindling and batch reset. This schedule enabled synchronized parturitions, efficient use of space and labor, and structured reproductive management—standard practice in commercial rabbit production.

Does were managed in cohorts synchronized on a 35-day cycle. Natural mating was employed, with directed mating practices: does were placed with selected bucks under supervision to ensure successful copulation and allow for individual parentage recording.

### 2.5. Housing, Feeding, and General Management

Does and bucks were housed individually in galvanized metal cages (90 × 60 × 40 cm) located in a naturally ventilated facility with thermal insulation in the roof and walls, and adjustable side windows. Cages were arranged in a flat-deck (single-row) system. Nest boxes were installed five days prior to the expected parturition date.

Each cage was equipped with an English-style hopper feeder (1.5 kg capacity) and an automatic nipple drinker providing freshwater ad libitum.

Animals were fed a commercial pelleted feed (Conejo, Unión^®^ Tepexpan, Acolman, Mexico). The analyzed chemical composition of the diet, on a dry matter basis, was as follows: crude protein, 15.75%; neutral detergent fiber, 49.84%; acid detergent fiber, 39.45%; ash, 8.45%; and organic matter, 91.55%. This formulation met the nutritional requirements for both maintenance and reproduction, supplying adequate levels of protein (12% and 15%, respectively) and fiber (10% and 14%, respectively), in accordance with NRC guidelines [30].

### 2.6. Sanitary Management

A comprehensive health management protocol was implemented on the farm to prevent and control common infectious and parasitic diseases affecting commercial rabbit production.

Bimonthly deworming was conducted using sulfonamides combined with trimethoprim (e.g., Koryn Triple) to control coccidiosis, a common parasitic disease in rabbits.

Multivitamin complexes (Ruviotic and Vitafort A) were administered to support metabolic function and reproductive performance.

Respiratory issues associated with cold weather or poor ventilation, which can lead to ammonia buildup, were managed by administering ambroxol and doxycycline (Valsyn) in the drinking water at a concentration of approximately 1 g doxycycline per liter, aiming to reduce secondary infections.

Mite infestations (sarcoptic mange) were treated topically with permethrin-based products (e.g., Bravo spray) following the manufacturer’s recommendations.

Diarrhea cases were treated orally with combinations of sulfonamides, trimethoprim, neomycin, and adsorbents such as kaolin–pectin formulations (e.g., Stop-One, Tomo, Kaofin).

Metabolic stimulants (e.g., Catosal) and vitamin A-D-E preparations (e.g., Vigantol) were used to enhance health and reproductive efficiency in breeding does. Vitamin dosages were carefully adjusted—typically ranging from 0.4 to 0.6 mL per doe—to avoid toxicity.

### 2.7. Ethical Considerations

All management practices and procedures described above reflect routine activities regularly conducted on the commercial rabbit farm. The detailed descriptions are provided solely to offer context for the research. No additional interventions were carried out on the animals specifically for this study. Therefore, ethical approval was not required, as all animals remained under standard farm care and management. The data analyzed were obtained retrospectively from farm records, accessed with permission from the farm management.

### 2.8. Data Editing and Inclusion/Exclusion Criteria

Records with missing or incomplete BR or WR values were excluded from the analysis. Stillborn and mummified kits were not included in BR. Relevant management events (e.g., feed reformulations, disease outbreaks, major infrastructure changes) were recorded and are discussed qualitatively in the Discussion. When doe or sire identification was missing, the record was retained for litter-level analyses but could not contribute to variance estimation for animal-level random effects.

### 2.9. Statistical Analysis

All analyses were performed using SAS 9.4 (SAS Institute Inc., Cary, NC, USA).

After verifying the normality of the variables through a normal quantile plot, a linear mixed model was fitted using PROC MIXED. The most comprehensive model was defined as follows:

$$Y_{ijk}\;=\;\mu \;+\;S_{i}\;+\;A_{j}\;+{\;E}_{k}\;+{\;\beta }_{1}{BR}_{l}\;+\;\varepsilon_{ijk}$$

where $Y_{ijk}$ = response variable (e.g., WR), *μ* = overall mean, *Sᵢ* = random effect of the *i*-th sire, *Aⱼ* = fixed effect of the *j*-th year of kindling, *Eₖ* = fixed effect of the *k*-th season of kindling, *β*_1_*BRₗ* = fixed effect of the *l*-th BR as a linear covariate (applied only to WR), and *ε_ijk_* = residual error.

### 2.10. Variance Components and Heritability

Variance components for sires ($\sigma_{sire}^{2}$) and the environment ($\sigma_{e}^{2}$) were estimated using the restricted maximum likelihood (REML) procedure. Least-squares means were computed and used for pairwise comparisons, applying the Tukey–Kramer adjustment. Statistical significance was declared at *p* < 0.05.

Heritabilities (h^2^) of the evaluated traits were estimated following Falconer and Mackay [31], using the following expression:

$$h^{2}=\frac{4\sigma_{sire}^{2}}{\sigma_{P}^{2}}$$

where $\sigma_{P}^{2}$ is the phenotypic variance, calculated as $\sigma_{P}^{2}=\sigma_{sire}^{2}+\sigma_{e}^{2}$, with $\sigma_{sire}^{2}$ representing the sire variance and $\sigma_{e}^{2}$ the environmental variance.

## 3. Results

The effects of year and season of kindling were significant for BR (*p* = 0.005; Table 1). The effect of year was also significant for WR (*p* = 0.021), whereas the effect of season on WR was not statistically significant (*p* = 0.152; Table 1).

**Figure 1:**
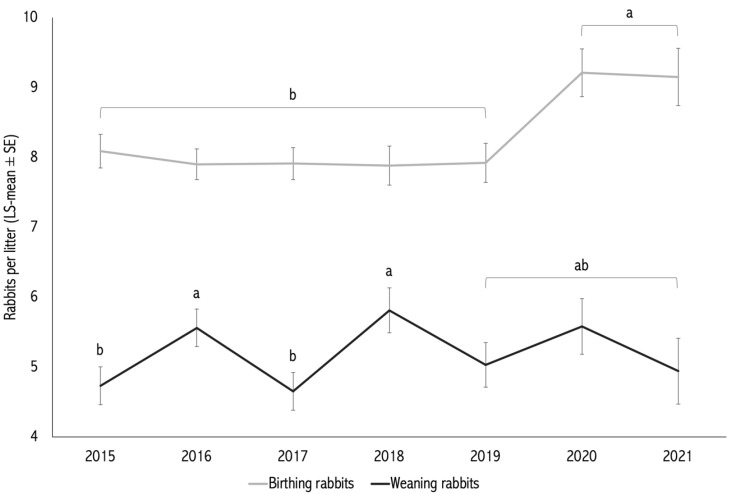
Annual trends (2015–2021) of adjusted means for number of kits born alive (BR) and number of kits weaned (WR). The effect of year was significant for both BR (*p* < 0.005) and WR (*p* < 0.021). Different letters (a,b) above the bars indicate statistically significant differences between seasons (*p* < 0.05).

Litters born in 2020 and 2021 had significantly higher BR than those born between 2015 and 2019 (*p* < 0.05; Figure 1). Regarding season, litters born during the semi-dry and humid periods (8.52 ± 0.18 and 8.56 ± 0.17 kits, respectively) had higher BR than those born in the dry period (7.80 ± 0.20 kits; *p* < 0.05; Table 1).

**Figure 2:**
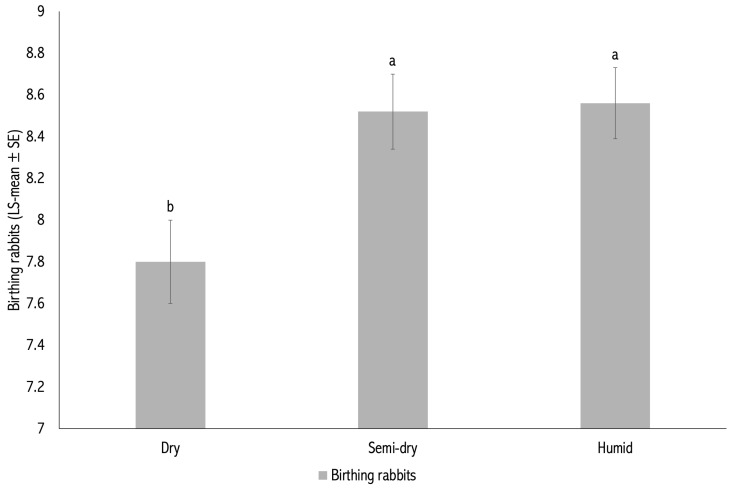
Number of kits born alive per litter (BR) by season of kindling. Different letters (a,b) above the bars indicate statistically significant differences between seasons (*p* < 0.05).

For WR, interannual differences were evident: the highest WR means were observed in 2016 (5.56 ± 0.27) and 2018 (5.81 ± 0.32), while other years showed intermediate or lower means (ranging from 4.65 to 5.58). Yearly weaning proportions ranged from 56.1% to 68.2%. Seasonal differences in WT did not reach statistical significance (*p* = 0.075), although the humid season showed the highest mean WT (65.49 ± 2.58%) (Figure 2).

Estimates of narrow-sense heritability were low under the current half-sib (sire component) model: BR h^2^ = 0.01, WR h^2^ = 0.04, and WT h^2^ = 0.05, indicating a marginal additive genetic contribution to these traits given the model and available data (Table 2).

## 4. Discussion

The results of the present study indicate that phenotypic variation in prolificacy (BR) was primarily driven by interannual and seasonal environmental factors (year and season effects), while the additive genetic contribution, as estimated from the sire variance component, was minimal. This pattern aligns with recent findings in rabbit production and other prolific species. In rabbits, thermal stress and seasonal management conditions negatively impact fertility, increase embryonic loss, and reduce neonatal survival, all of which directly influence the number of live-born kits [27]. Recent studies have reported clear physiological effects of heat on female fertility and male semen quality, as well as hormonal and embryonic disruptions that reduce litter size during and following periods of heat stress [32]. These mechanisms largely explain why the dry season, characterized by more severe environmental stress, is associated with lower BR compared to more favorable climatic periods.

The higher prolificacy observed in 2020–2021 compared to previous years may reflect interannual climatic variation—such as more frequent thermally favorable conditions or fewer extreme events—that temporarily improved doe body condition and fertility [33]. Alternatively, changes in management practices during those years (e.g., nutrition, health protocols, reproductive strategies, or adjustments in batch structure) may have contributed to the observed improvement [34]. Similar combinations of climatic and management-related factors have been reported in other studies to explain interannual variation in reproductive performance [32,33].

The seasonal effect observed can be interpreted considering the interaction among temperature, humidity, and nutritional availability. Under extreme dry and high heat conditions, voluntary feed intake declines, body condition deteriorates, and thermal stress increases—factors that compromise ovulation, early gestation, and early lactation, ultimately leading to smaller litters or increased embryonic mortality [27]. In contrast, temperate conditions with greater feed availability tend to support improved body condition and reproductive performance.

The rabbit’s response to heat stress is complex and non-linear. For example, moderate humidity under non-extreme temperatures may be less detrimental than drought conditions accompanied by feed scarcity—consistent with the patterns observed in this population [4]. These physiological and management-related interactions with the environment are well documented in recent reviews on the effects of heat stress on rabbit reproduction and performance [13,32].

Regarding WR and WT, our results showed a significant year effect for WR, but no statistically significant seasonal effect; WT also did not differ significantly by season. This dissociation supports the idea that pre-weaning survival is strongly influenced by maternal and postnatal management factors—such as housing quality, perinatal care, nutritional supplementation, weaning practices, and herd health—rather than by the season of birth per se [17].

Recent studies indicate that successful weaning depends largely on maternal ability, neonatal management, and litter microenvironment—factors that, when adequately managed, can stabilize survival outcomes across seasons [34,35]. Therefore, the absence of a strong seasonal effect on WR and WT suggests that targeted perinatal interventions—including biosecurity measures, litter management, local temperature control, and nutritional support—are crucial for improving survival rates [36,37].

Moreover, a more comprehensive recording of mortality etiology could enhance survival analysis by contextualizing the observed seasonal and annual variation in the evaluated traits.

Estimated heritabilities were very low, consistent with previous reports of reduced values for reproductive traits measured at the litter level [37]. Litter traits are influenced by a combination of factors, including direct additive genetic effects, permanent maternal effects, temporary environmental influences, and management practices—of which the additive genetic contribution is often minimal [38]. Recent studies in rabbits and other species confirm that h^2^ estimates for litter traits tend to be low, and that the use of more comprehensive models—such as animal models incorporating permanent maternal permanent effects—can improve parameter estimation and, in some cases, increase the estimated additive component [39].

Consequently, given the low h^2^ values observed in this study and the available experimental design, the expected response to genetic selection would be very limited. However, for WR, some genetic progress may still be achievable. Improving the accuracy of doe identification and litter recordkeeping would enhance the genetic characterization of the evaluated traits, enabling the inclusion of maternal and maternal environmental effects in the models and providing a more reliable prognosis for genetic selection response.

## 5. Conclusions

This study demonstrated that both year and season of kindling significantly influenced reproductive performance in rabbits raised under subtropical–semi-dry conditions in central Mexico. Seasonal fluctuations in temperature and humidity were associated with variations in litter size at birth and weaning, confirming the high environmental sensitivity of rabbit prolificacy. These findings underscore the importance of implementing environmental and thermal management strategies to sustain productivity during climatically unfavorable periods.

The low heritability estimates for litter traits suggest that genetic improvement through selection alone is limited, as environmental and maternal effects play a predominant role. Therefore, enhancing reproductive performance will depend primarily on improved environmental control, nutrition, and health management, alongside genetic programs aimed at increasing resilience and maternal ability.

Overall, this study provides baseline information on climate-related reproductive responses in rabbits under subtropical conditions, contributing to the development of more sustainable and adaptable production systems. Future research incorporating climatic indices and genomic tools could further elucidate genotype–environment interactions affecting prolificacy and survival.

## Figures and Tables

**Table 1:** Least square means (± standard error) for litter size at birth (BR) and weaning (WR), and weaning rate (WT) of rabbits in Central Mexico by year and season of kindling.

Item	n	BR	WR	WT (%) ^1^
Year				
2015	145	8.09 ± 0.24 ^b^	4.73 ± 0.27 ^b^	57.09 ± 3.49
2016	155	7.90 ± 0.22 ^b^	5.56 ± 0.27 ^a^	65.91 ± 4.42
2017	154	7.91 ± 0.23 ^b^	4.65 ± 0.27 ^b^	56.09 ± 3.49
2018	100	7.88 ± 0.28 ^b^	5.81 ± 0.32 ^a^	68.17 ± 4.14
2019	101	7.92 ± 0.28 ^b^	5.03 ± 0.32 ^ab^	60.83 ± 3.99
2020	67	9.21 ± 0.34 ^a^	5.58 ± 0.40 ^ab^	66.57 ± 5.05
2021	48	9.15 ± 0.41 ^a^	4.94 ± 0.47 ^ab^	61.32 ± 5.87
*p*-value (year)		0.005	0.021	0.107
Season				
Dry	202	7.80 ± 0.20 ^b^	5.37 ± 0.24	63.32 ± 2.97 ^ab^
Semi-dry	255	8.52 ± 0.18 ^a^	4.87 ± 0.20	58.05 ± 2.64 ^b^
Humid	313	8.56 ± 0.17 ^a^	5.31 ± 0.20	65.49 ± 2.58 ^a^
*p*-value (season)		0.005	0.152	0.075

^1^ Weaning rate calculated as WT = (WR/BR) × 100; ^a,b^ Means within a column with different superscript differ significantly (*p* < 0.05).

**Table 2:** Estimated heritabilities (h^2^) for litter size at birth (BR), litter size at weaning (WR), and weaning rate (WT) obtained from a sire model.

Trait	h^2^ ^1^	SE	Interpretation
BR (Born alive per litter)	0.01	0.01	Very low additive genetic influence; dominated by environmental variation
WR (Weaned per litter)	0.04	0.02	Low heritability; minor additive component
WT (Weaning rate)	0.05	0.02	Low heritability; marginal potential for selection response

^1^ Estimates derived from sire variance components in a one-way random effects model using REML procedures.

## Data Availability

The original contributions presented in this study are included in the article. Further inquiries can be directed to the corresponding author(s).

## Abbreviations

The following abbreviations are used in this manuscript:

BR	Number of kits born alive per litter
WR	Number of kits weaned per litter
WT	Weaning rate, calculated as WT = (WR/BR) × 100
P	Probability
h^2^	Heritability
REML	Restricted maximum likelihood
